# Personalized paths for physical activity: developing a person-centered quantitative function to determine a customized amount of exercise and enhancing individual commitment

**DOI:** 10.1186/s13102-021-00282-4

**Published:** 2021-06-02

**Authors:** Giovanni Iolascon, Francesca Gimigliano, Gioconda Di Pietro, Antimo Moretti, Marco Paoletta, Matteo Rivezzi, Alessandro Distante, Prisco Piscitelli

**Affiliations:** 1 Department of Medical and Surgical Specialties and Dentistry, University of Campania “Luigi Vanvitelli”, Naples, Italy; 2 Department of Mental and Physical Health and Preventive Medicine, University of Campania “Luigi Vanvitelli”, Naples, Italy; 3 Doctorate in Translational Medicine, University of Foggia, Foggia, Italy; 4 Euro Mediterranean Scientific Biomedical Institute, ISBEM, Bruxelles, Belgium; 5 Staff UNESCO Chair on Health Education and Sustainable Development, Federico II University of Naples, Naples, Italy

**Keywords:** Physical activity, Exercise, Personalized approach, Healthy lifestyle, Metabolic equivalents

## Abstract

**Background:**

Non-Communicable Diseases (NCDs) are leading causes of mortality. These conditions are also known as chronic diseases of long duration and generally slow progression. Physical activity (PA) is a main factor to delay symptoms and consequences of NCDs. In last decades, reduced physical exercise has been observed across all ages. Despite educational campaigns aimed at modifying unhealthy habits, it is difficult to promote healthy lifestyles in general population. Poor interest, lack of motivation, as well as career and family commitments hinder people’s participation in regular PA programs. In this study we propose a theoretical person-centred approach to actively involve general population in enhancing their opportunity to perform PA based on personalized needs and targets.

**Methods:**

We defined four profiles of baseline PA levels (inactive, moderately inactive, moderately active, and active people) by referring to Metabolic equivalents (METs) based on individual answers to General Practice Physical Activity Questionnaire (GPPAQ).

**Results:**

Based on the answers to the GPPAQ and by computing the related METs for each profile of baseline exercise levels, we developed an innovative person-centered web-based algorithm/function for enhancing and measuring PA participation in community settings. This function can compute evidence-based standardized profiles of participants, personalized goals of PA being functional to the purpose of maintaining or gaining health benefits, as well as the type and duration of PA needed to reach these goals.

**Conclusion:**

It might be speculated that this approach would be a reliable method for increasing people’s self-efficacy and population adherence to recommended levels of PA. However, this theoretical proposal requires to be implemented in further research.

## Background

The epidemiological explosion of Non-Communicable Diseases (NCDs) is one of the main concerns of the health systems representing the leading causes of annual deaths worldwide [1].

Non-Communicable Diseases, also known as chronic diseases, affect many adults and are characterized by slow and age-associated progression. Despite a higher prevalence of these conditions in older populations, surprisingly over 9 million deaths attributable to NCDs, such as cardiovascular and respiratory diseases, type 2 diabetes, and cancers, occur in younger people, without significant difference between men and women [2]. Most risk factors for NCDs could be modified through effective interventions, particularly addressing tobacco use, unhealthy diet, and physical inactivity [3]. Educational campaigns of both governments and non-governmental organisations aiming to modify unhealthy habits in general population have a limited impact on entrenched habits [4]. Indeed, it is historically known that people have serious difficulties in defining, understanding, and adopting healthy lifestyles, even in those cases in which they are aware of the detrimental consequences deriving from the non-observance of such behaviours [5].

In this context, WHO supports a new concept based on people empowerment, shifting from a delivered healthcare model to a person-centred approach where the stakeholder is actively involved in maintenance or improvement of their health status, such as increasing levels of physical activity (PA) [6]. Even if beneficial effects of exercise have been widely documented, in the last decade a worrying decline in PA practice has been observed across all ages, including even children and adolescents. For example, it has been estimated that on a typical week, 40% of adults in Europe engaged in no exercise or sports [7]. Among the main reasons behind these data is that around 40% of Europeans report that they have not enough time to be engaged in exercise and 20% of them report that they are not interested in exercise at all and that they prefer to spend their time to do other activities [7]. Physical activity is not a priority due to social, cultural, and economic reason: physical activity costs money, the rewards are often unclear to the public. There is no strong advice from the health system and policy makers. For example, if physical activity will be covered by health insurance, it is likely that many more people will participate in such activities. If physicians will advise physical activities to NCDs patients, and a progress will be monitored it is expected that those patients will follow these recommendations.

To enhance people insights about effectively promotion of a healthy lifestyle, is mandatory to define different types of PA [8]. This is defined as any movement of the body resulting from the contraction of skeletal muscles that increases energy expenditure above a basal level. Physical activity can be divided in unstructured (PA incorporated in daily life) or structured, defined as exercise that is a PA planned and repetitive. Therefore, PA includes also occupational, household as well as leisure-time activities [9]. Other environmental prevention strategies that may enhance participation in regular PA is to plan and conduct successful communication campaigns as well as to increase the opportunity to be involved in this activity [1]. This study was aimed at defining standardised profiles of recommended PA and to develop both personalized goals for general population, considering the needs of each category of people for increasing or maintaining the level PA, as well as the algorithms (type and duration of exercise) needed to reach these goals.

## Methods

The standardized profiles and the personalized goals have been defined on the basis of the WHO guidelines for PA [10]. The level of PA has been defined using the General Practice Physical Activity Questionnaire (GPPAQ) [11] to quantify Metabolic equivalents (METs) performed in a week. The METs have been used to define the personalized goals.

The proposed model is designed to run through Java Script software that preliminarily takes into account age, gender and any disease of users that may limit the practice of PA before accessing the algorithm. It uses Entrez Programming Utilities to send search queries, retrieve results in XML format and display the physical exercise list. Once the search results are loaded, dynamic HTML, DOM tree manipulation and Ajax scripting transforms the static page into an interactive application. Common Web standards were adopted during script coding and the application should work in standards-compliant Web browsers. In case some individuals are affected by one or more diseases, these conditions must not reduce the independence in the activities of daily life, nor require any type of pharmacological treatment. To take advantage of using this innovative web-based application, a basic computer literacy is required, because the algorithm is designed to work in Windows and developed to work in a very intuitive environment, including visual selections. To further improve the compliance to the use of this tool, initial instructing for the users will be provided. Therefore, advanced IT skills are not required.

### Definition of standardized profiles of recommended physical activity level

We have defined the following standard of PA, namely the volume of PA, expressed in METs, being functional to the purpose of gaining health benefits: an overall volume of weekly PA (3–5 times/week) to meet a minimum of 150 min of moderate-intensity activities or 75 min of vigorous-intensity activities (150*3.0–5.9 MET = 450–885 METs or 75*6 or more = 450 or more METs) up to 300 min of moderate-intensity activities or 150 min of vigorous-intensity activities (300*3.0–5.9 MET = 900–1770 METs or 150*6 or more = 900 or more METs), regardless of gender and age.

### Definition of different individual goals of physical activity

The proposal of a goal of PA to the user will make based on the following tenets.

1. The user will be classified in accordance with the level of current PA. The user will be advised to adopt the defined goal according to the type of person the user has been classified (i.e., people having the same level of activity take advantage by assuming “X” as level of PA).
2. The standard will be hierarchically differentiated – namely a distinction between **basic** and advanced standard will be put. Basic goal is the amount of PA (900 METs) associated with substantial health benefits. Advanced goal is the amount of PA (1800 METs) associated with additional health benefits. Each user will be asked to choose the level of goal to be assumed as own purpose (needless to say, s/he will have the chance to update it over the time).
3. A dynamic architecture of goals has been designed, namely an architecture that considers the reaching of a certain goal for the adoption of a further, more advanced goal. Thanks to such a dynamic architecture, it will be possible to define incremental path enabling user to adopt intermediate goals being more consistent with their condition and to take advantage from the motivating effect of the experience of reaching and keeping what they define as goal. It is worth highlighting the conceptual difference with the distinction proposed in the previous point. In this case the different amount of PA is put at the service of the design of an incremental plan of activity; instead, the distinction considered in the previous point concerns the absolute level of PA (and therefore of health advantage) the user chose as own standard.

### Segmentation of users and identification of prototypical models of activity

Users will be segmented in accordance with their current volume of PA, esteemed by means of the self-report GPPAQ [11]. To this end will be adopted the following 4 profiles of current activity, based on the GPPAQ:

- Inactive people
- Moderately inactive people
- Moderately active people
- Active people

Each segment will be associated with a *prototypical model of activity* (see Table 1), defining:

- One or two standards of weekly volume of activity (basic and/or gold), and.
- An appropriate progressive path of pursuing.

**Table 1:** Users’ segmentation and prototypical models of activity

Profiles of users	Basic standard	Gold standard	Progressive paths of pursuing (week 1, 2, 3, 4, 5–8; percentage of coverage of the standard chosen)
Inactive	> 450 METs	**–**	(Try and check) 0.3/0.5/0.5/0.75/1
Moderately inactive	> 450 METs	900 METs	(Diesel engine) 0.5/0.75/0.75/1/1
Moderately active	900 METs	1800 METs	(Two step) 0.75/0.75/1/1/1
Active	–	1800 METs	(Speedy Gonzalez) 0.75/1/1/1/1 (All in one) 1/1/1/1/1

Each profile is associated to a prototypical model of activity, including different weekly volumes of physical activity to be performed, as well as the mode of progression (indicated by idioms that facilitate the identification of the type of user, including the percentages of coverage of the goal of physical activity) to reach the desired goal

*Abbreviation: METs* Metabolic equivalents

At the moment, as first stage of development of the model, we have planned to define 5 progressive paths, easily identifiable in their rationale and content, in order to make them both immediately understandable by users and available to be modeled at the computational level. Each progressive path will be marked by an image synthesizing its functional meaning and therefore facilitating the identification of the type of user fitting it (i.e., “Speedy Gonzales”; “Two Step”; “Diesel engine”, the “Try and check”, “All in one”).

### Procedure of goals and progressive program setting

Each user can be asked to fulfill the GPPAQ on a web-based platform. The platform will make it explicit the profile of current activity (among the possible 4) the user will be resulted classified. Basic pockets of evidence-based knowledge will be provided to highlight the risks/benefits associated with the profiles.

**Fig. 1:**
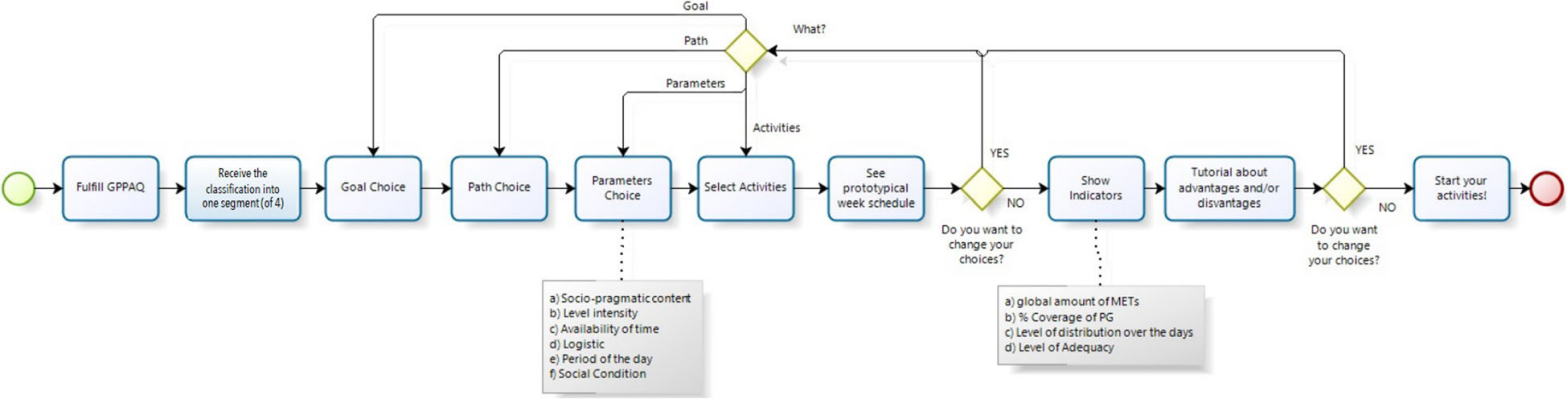
The procedure of the physical activity planning. A global look

**Fig. 2:**
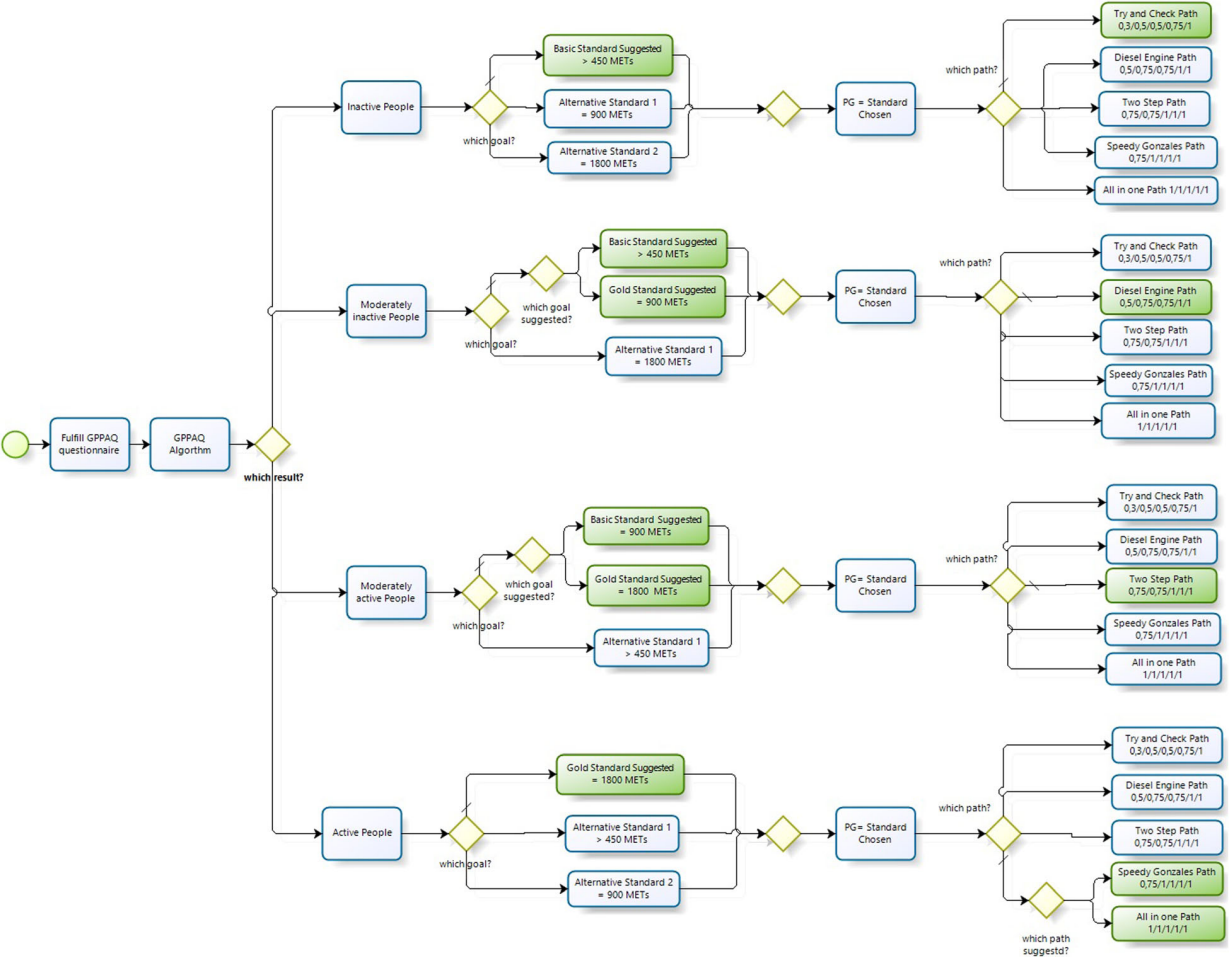
The procedure of goal and path setting *(Prototypical paths and goals in green)*

Then, the user is asked to confirm his/her intention to design a program of PA. If the user accepts, the procedure of goal and path starts. Figures 1 and 2 detect the workflow of the procedure.

First, the user will be advised as to the standard(s) associated with the segment s/he has been classified. However, the advices will not be normative, in the sense that they will be provided in terms of suggestions rather than imposed to users. Thus, the chance to choose a standard (and a progressive path) by one’s own will be left to every user.

Once the client will have confirmed or changed the standard, this will be the Personal Goal (PG). Thus: *PG = St*, where *PG* stands for *Personal Goal*, *St* for the chosen *Standard Goal*.

Second, the user will be provided with a default progressive path, the one associated with the segment (according to the scheme reported in Table 1). Yet, it will be highlighted that s/he has the chance to change it if s/he should find it preferable a different progressive path (or the all-in-one path). A brief textual and graphical presentation of each path, with the associated rationale will be provided, to make it explicit which kind of need and attitude it fits with. Standard, goals, and paths will be provided both in terms of METs and amount of time.

Also, after having started the program, the user will have the possibility to change goal and path at any time. The new program will start always on the nearest Monday. Incidentally, as further development, it will be worth enabling the system to provide the suggested standard and progressive path as function of the user’s socio-psychological profile as well as socio-ecological scenario she/he is part of.

## Results

### The selection of the content of physical activities

Once goal and progressive path are set, the user will be asked to choose the content of the physical activities, to build her/his program.

To this end, a dataset of blocks of activity has been defined. Each block is a 10-min unit of PA (e.g., housing, washing glasses, walking) associated with the corresponding METs.

The user will be asked to choose the blocks of activity fitting with his/her constraints/preferences. The user will do so by selecting characteristics of activities as defined by a set of parameters. The selection of a certain characteristic will correspond to the deactivation of a filter operating upon the dataset of blocks. The parameters are the following:

- the *content of the activity* (e.g., sport activity, leisure, gardening, housing, and so forth).
- the level of intensity of the activity (low-moderate-high).
- the *user’s availability of time* (high – i.e., HIGH: more than 3 h daily/MIDDLE: 1–3 h/LIMITED: less than 1 h).
- the *venue of the activity* (indoor/outdoor/both).
- the *moment of the day* related with the activity (morning/afternoon/evening/not depending on the moment of day).
- the *social condition of the activity* (alone/requiring at least a couple/requiring a team).

User will be able to select all aspects s/he wants, in order to personalize own set of blocks of activity fitting with own project.

Blocks of activity will be classified in accordance with each parameter (Table 2 provides an exemplificative excerpt of the classification of activity used for this purpose). In this way, the user’s activation of one parameter will correspond to the selection of only the blocks tagged by those modalities and the deselection of others.

**Table 2:** Excerpt of the classification of activity

Block Activity			Parameters															
Content	Name	MET value (for 1 min)	Level			Time			Venue			Moment of day				Social Condition		
			***Low***	***Moderate***	***High***	***High (> 3 h/d)***	***Middle (1–3 h/d)***	***Limited (< 1 h/d)***	***Indoor***	***Outdoor***	***Both***	***Morning***	***Afternoon***	***Evening***	***Not depending***	***Alone***	***Couple***	***Team***
Household Activities	Interior Cleaning	3.01	No	Yes	No	No	No	Yes	Yes	No	No	No	No	No	Yes	Yes	No	No
Household Activities	Laundry	2.07	Yes	No	No	No	No	Yes	Yes	No	No	No	No	No	Yes	Yes	No	No
Household Activities	Sewing, repairing, and maintaining textiles	1.5	Yes	No	No	No	Yes	No	Yes	No	No	No	No	No	Yes	Yes	No	No
Household Activities	Storing interior HH items, including food	3.39	No	Yes	No	No	No	Yes	Yes	No	No	No	No	No	Yes	Yes	No	No
Household Activities	Housework, n.e.c.	2.51	Yes	No	No	No	No	Yes	No	No	Yes	No	No	No	Yes	Yes	No	No
Household Activities	Food and drink preparation	2.16	Yes	No	No	No	No	Yes	No	No	Yes	No	No	No	Yes	Yes	No	No
Household Activities	Food presentation	2.38	Yes	No	No	No	No	Yes	Yes	No	No	No	No	No	Yes	Yes	No	No
Household Activities	Kitchen and Food Clean-Up	2.54	Yes	No	No	No	No	Yes	Yes	No	No	No	No	No	Yes	Yes	No	No

Thus, eventually, the platform will provide the user of the *fitting blocks of activity***,** namely the ones corresponding to her/him condition and preferences (i.e., her/his project).

It is worth adding that, for every expected combination, the dataset will hold blocks of activity which one can expect are able to meet the need of persons of different socio-economic level as well as gender. Moreover, blocks for people with special need will be provided. This will be so because these sources of variability in the users’ preference are associated with sensible data. Therefore, not being possible to ask data about them directly to users, such sources will be considered downhill, by providing the user with a set of alternatives encompassing them.

### The week planning of the activity

The blocks of activity selected in the previous step will work as the “Lego” units in the following step, devoted to the building of the weekly schedule of the personalized program of PA.

On one side of the screen the user will find the blocks of activity s/he has selected (*fitting blocks*). Form and color of blocks will indicate their main characteristics (e.g., form for the type of activity and color for the level of intensity). Information concerning other parameters will be provided by means of the block position on the screen. Moreover, each block will report the corresponding METs inside.

On the other side of the screen, the user will find an empty week agenda plan (segmented in units of 10 min) to be used for distributing the fitting blocks over the week.

User will be invited to plan her/his own schedule, placing the blocks within the week agenda plan, taking the block from the set of fitting blocks exposed on the other side of the screen.

**Fig. 3:**
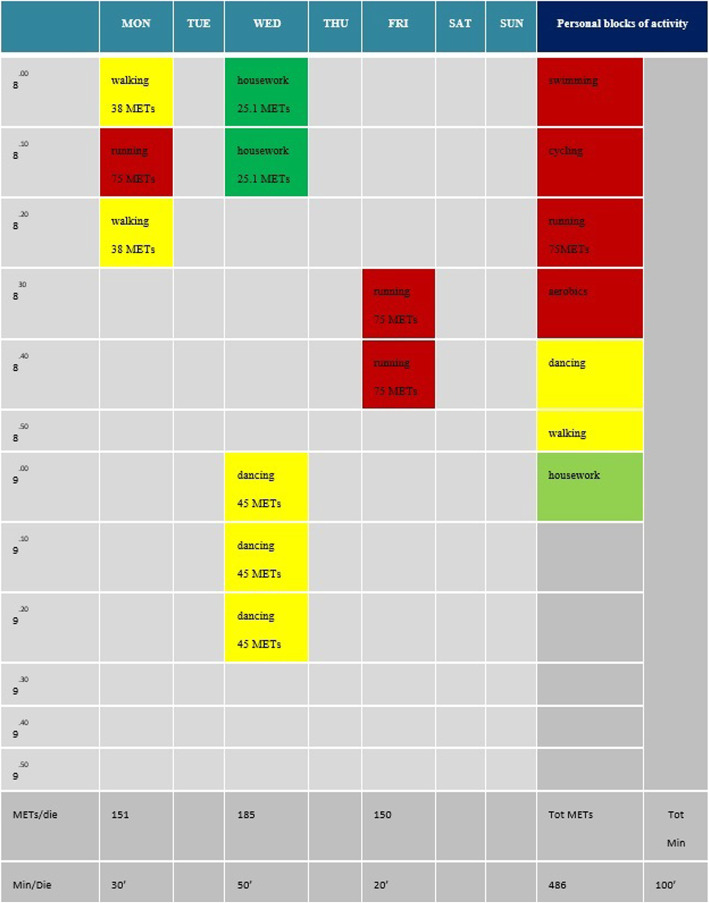
An example of week schedule of physical activity

Figure 3 provides an example of week plan of the PA.

In parallel with the planning, the platform will provide indicators of the level of consistency of the schedule with the Personalized Goal. More in particular, the following indexes will be provided:

A) *The global amount of MET set over the week* (*GA*_*w*_). Thus, *GA*_*w*_ represents the total amount of METs corresponding to the blocks scheduled.

*GA*_*w*_ is calculated in the following way:

$$ {\mathrm{GA}}_{\mathrm{w}}={\sum}_{i=1}^7{\sum}_{k=1}^n{B}_{ik} $$

Where *B* is the METs distributed over the week, i represent the generic day in a week, n is the number of activity.

B) *The level of coverage for the week (CV*_*w*_*),* namely the ratio in percentage of GA_w_ out the Personal Goal for that week (as defined by the path chosen). *CV*_*w*_ can vary from 0 to 100%. The maximum *CV*_*w*_ is given when the MET scheduled in the week (*GA*_*w*_) are the ones foreseen by the path chosen. If *GA*_*w*_ corresponds to the Personal Goal, *CV*_*w*_ will be 100%.

*CV*_*w*_ is calculated in the following way:

$$ {\mathrm{CV}}_{\mathrm{w}}=100\ast {\mathrm{GA}}_{\mathrm{w}}/{\mathrm{PG}}_{\mathrm{w}} $$

Where PG_w_ is the Personal Goal of the week, as defined by the path chosen

III) The *global distribution of the MET* over the week (*D*_*w*_). This index esteems the homogeneity/concentration of the activity across the days of the week.

*D*_*w*_ is calculated in the following way:

$$ D=\frac{\operatorname{MAX}}{\operatorname{MIN}} $$

where MAX is the amount of METs planned in the day of the week with the maximum amount of daily METs; MIN is the amount of METs planned in the day in the week with minimum amount of daily METs.

*D*_*w*_ will be expected not to be higher than 1,75. In formulas:

$$ D\le 1,75 $$

This means the MAX value should not be higher than the double of MIN. This is so for the sake of defining a criterion guiding the user to schedule the activities (and relative METs) homogeneously over the week.

IV) The *fitness of the distribution* (*F*_*w*_) respect on the basic distribution suggested by literature (i.e. METs distributed over 3 days). This index esteems the homogeneity of the distribution of METs over the week. This will be done in terms of the computation of how many days of the week present an amount of METs scheduled that is over the level of the standard concentration.

*F*_*w*_ is calculated in the following way.

First, the level of standard concentration (CONw) is defined, in the following way.

$$ {CON}_w=\left({GA}_w/3\right)+0,2\left({GA}_w/ 3\right) $$

Thus, the level of concentration is given by one third of the amount of MET scheduled for the week (*GA*_*w*_), increased of 20% of it.

Then, *F*_*w*_ is given by the number of days having METs higher than *CON*_*w*_.

A tutorial will highlight advantages and/or disadvantages for each index and (where being the case) suggestions to improve the functionality of the schedule.

### Self-monitoring

The user will be asked to check the scheduled activities daily, accordingly to a 4 point scale (“I did more!”; “I did them!”; “I did them partially”; “I did not them”) associated with a corresponding icon (e.g. a smile, a sorrow face). Each day will be classified accordingly (namely, as a “I did more!” day, a “I did them” day and so forth)

In order to calculate the indexes indicated below, the modality “I did more!” is considered conventionally being equivalent to 1.25 times the scheduled METs of the day. The modality “I did them partially” is considered conventionally being equivalent to 0.5 time the scheduled METs of the day.

In the event the user skips the daily monitoring, the first time s/he accesses newly to the platform, s/he will be asked to check the activity not monitored before going on. More in particular, the user will be asked if the not monitored activities must be considered not accomplished. If the user will answer “Yes”, all not monitored activities will be classified accordingly, as “I did not”. If the user will answer “No”, s/he will be asked to check them day-by-day.

### Day and week feedback

The system will provide a daily and a weekly feedback, respectively at the end of the day and of the week.

### Daily feedback

The daily feedback will be aimed at reinforcing the user in the case s/he has accomplished the daily task or to motivate/support her/him in completing it in the following day(s).

**Fig. 4:**
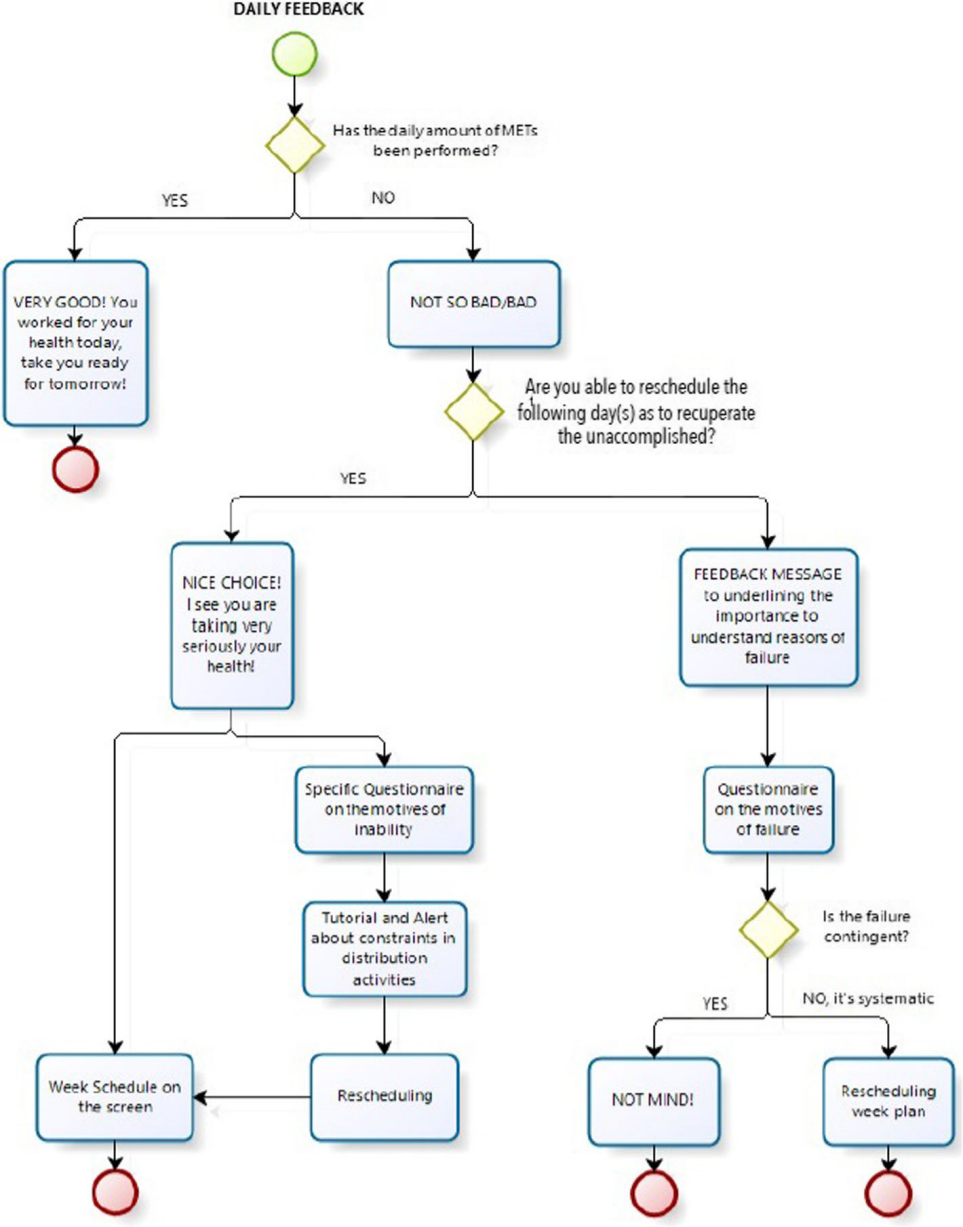
Decisional tree of the daily feedback

To this aim, a synthetic feedback will be provided, according to the following decisional tree (see Fig. 4).

A) if the daily amount of METs, or more, has been performed, the system will provide a positive feedback (e.g., *“Very good- you worked for your health today! Take you ready for tomorrow”*) to the user.
B) if the daily amount of METs has not been reached, the user will be asked if s/he intends/is able to reschedule the following day(s) to recuperate the unaccomplished amount of METs.
  B1) if the user answers “Yes”, the system will provide a reinforce message (e.g., *“nice choice; I see you are taking very seriously your health!”*) and the week schedule will appear on the screen, together with a multiple-choice questionnaire on the motives of the inability to accomplish the daily activities. Moreover, a tutorial will support the rescheduling of the week plan, providing one or more appropriate simple strategies for recuperation (e.g. to change duration and/or intensity of activity; to add new activity; to displace the task in another moment of the day); finally, the tutorial will warn as to the constraints concerning the distribution of the activities over the week - this in order to avoid that the rescheduling could produce an unhealthy distribution of METs on the week. Incidentally, it is worth noting a line of development of the system entailed in the architecture outlined above – joining information obtained by the questionnaire and by the rescheduling (the former provided by the user, the latter extracted by the system by means of the computation of the difference between schedule and reschedule) will be possible to identifying profiles of user characterized by specific matching between motives of failure and strategies of facing them.
  B2) In the case the user answers “No”, the system will provide a support message, underlining that what is important is to understand the reasons of the missed accomplishment, to find the fittest week schedule. Also, in this case, the user will be asked to fulfill the questionnaire on the motives of failure (the same of B1). Moreover, s/he will be advised to check if the failure has to be interpreted as contingent or systematic (i.e., if contingent, then no mind; if it is the effect of a systematic motive, then the user will be invited to revise the following part of the week plan).

### Weekly feedback

The weekly feedback concerns the appropriateness of the schedule for the user. It is provided at the end of the week and it is aimed at checking the validity of the schedule (i.e., its appropriateness for the user) and/or to modify-modulate it in order to increase its validity for the user.

### Weekly report

A week report of the activity will be produced. This report will be based on the daily self-monitoring and will provide the following summary statistics:

1. the amount of METs performed in the week (*PERF*_*w*_*)*;
2. the distribution of METs over the week (Monday, Tuesday...) and previous weeks (i.e., week 1, 2);
3. the distribution of METs over the fitting blocks of activity;
4. the distribution of METs over categories of activity (the ones identified by parameters: e.g., content, venue and so forth);
5. the distribution of the 4 type of days (“I did more!”, “I did them”) over the last 4 weeks;
6. the distribution of the type of days (“I did more!”, “I did them”) over the last 4 weeks differentiated for categories of activity (the ones identified by parameters).

### Weekly feedback

**Fig. 5:**
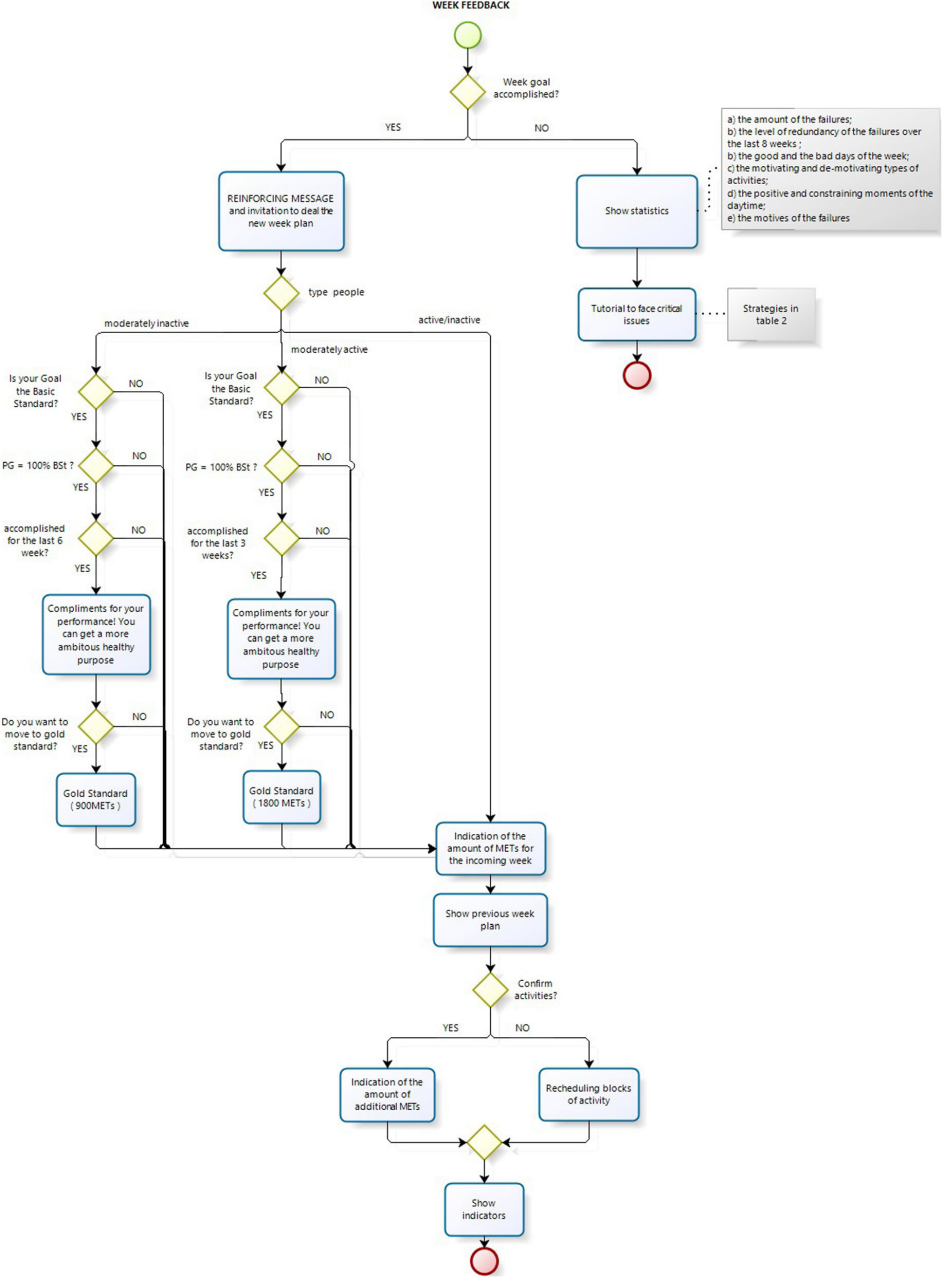
Decisional tree of the week feedback

A synthetic feedback will be provided, according to the following decisional tree (see Fig. 5**).**

#### Condition 1. Week success

If the user has accomplished the personalized goal of the week, then s/he will receive a reinforcing message and the invitation to deal with the new week plan.

The user will be provided with the indication of the amount of METs defined by her/his progressive path for the incoming week. S/he will be asked to confirm the chosen PG for the incoming week and to schedule the activities over the week accordingly.

In the case of users classified as “moderately inactive” and “moderately active”, who had chosen the basic standard, if their personalized goal corresponds to the 100% of the basic standard and if their personalized goal has been accomplishing systematically for the last - respectively - 6 and 3 weeks, then they are proposed to move to the gold standard (1800 METs). To this end, a message of compliments will be appearing on the screen, underlining the value of the performance and the chance to get a more ambitious, healthy purpose.

In order to support the user’s rescheduling, the previous week plan will appear on the screen, in the case with the indication of the amount of additional METs required as well as the possibility of revising the types of blocks of activity selected the week before. Indicators described in paragraph 6 (e.g., *GA*_*w*_, the global amount of METs scheduled for the week) will be provided as well.

#### Condition 2. Week failure

If the week goal has not been accomplished, the system will calculate a set of statistics to provide a framework supporting the user’s decision making.

Statistics will concern:

- the relative amount of METs not accomplished in the current week respect on the scheduled METs (*UNC*_*w*_). *UNC*_*w*_ is computed as the difference between the scheduled METs for the week and the METs accomplished divided the scheduled METs. In formulas:

$$ {\mathrm{UNC}}_{\mathrm{w}}=100\ast \left({\mathrm{GA}}_{\mathrm{w}}-{\mathrm{PERF}}_{\mathrm{w}\Big)}\right)/{\mathrm{GA}}_{\mathrm{w}}; $$

b) the *UNC*_*w*_ concerning the previous weeks of the program;
c) the *good* (“I did more” and “I did them”) and the *bad* (“I did them only partially” and I did not them”) days of the week;
d) the distribution of the *UNC*_*w*_ over the types of activity (e.g., between content; between moment of daytime, venue and so forth);
e) the frequency motives of the failures identified by the questionnaire (both global statistics and disaggregated for days and types of activity)

A tutorial will be proposed to the users, with the advices concerning the ways of facing the critical issues highlighted by statistics a-e. To this end, the numeric values will be presented in ordinal scale too. More specifically,

- *UNC*_*w*_ < 20 “this aspect is not problematic”;
- *21 < UNC*_*w*_ < 39; “this aspect is moderately problematic”;
- *UNC*_*w*_ > 40“this aspect is highly problematic”.

The advices will be organized in hierarchical way, moving from the proposal of strategies (among the ones functional to the critical issues associated with the user) having the lowest impact on the plan to the one having the highest.

The personalized combination of the advices will be calculated by means of the matrix *critical issues-strategies for facing them*. For each critical issue (encompassing both the detection of failures and the motives identified by the user) a pertinent set of strategies will be proposed.

## Discussion

In the perspective of reducing the impact of NCDs, we have developed an innovative web-based person-centred quantitative function to foster individual motivation towards physical exercise. Based on the GPPAQ and by computing the METs corresponding to each profile of exercise levels, we have set a quantitative function able to set individual goals and paths.

Physical activity has been defined by WHO as “any bodily movement produced by skeletal muscles that requires energy expenditure” [9]. It is important to take in mind this definition when discussing on how to define its assessment. There are many available subjective (self-report questionnaires) and objective (indirect calorimetry, direct observation, heart rate telemetry, and movement sensors) methods to assess PA. All of them have well-known limitations. Movement sensors such as accelerometers are quite popular being able to give an objective measure of PA and being of relatively small size, however, due to their high costs, they are not usually practical in large-scale cohort studies where questionnaires are preferred [12]. Recent reviews have documented up to 85 self-administered PA questionnaires for adults [13]. The most important features of a self-administered questionnaire are simplicity, cultural equivalency (international comparability), repeatability, construct validity, sensitivity and specificity, reliability, economy. Considering all these characteristics, the most suitable PA questionnaire, for the purpose of our project, is the GPPAQ. This is a reliable and validated tool to assess adult level of PA for use within primary care and its use is supported by the National Institute for Health and Care Excellence (NICE) [14]. To complete the GPPAQ only 60 s are necessary. It generates a simple, 4-level PA index, categorizing patients as active, moderately active, moderately inactive, or inactive. Therefore, we endorsed the use of GPPAQ for the purposes of our project.

Our approach allows to define evidence-based standardised profiles, personalized goals of PA being functional to the purpose of maintaining or gaining health benefits, as well as the algorithms (type and duration of PA) needed to reach these goals.

Available evidence about the effectiveness of lifestyle intervention, including regular PA, for the prevention and management of several NCDs [3], does not correspond to an adequate effort in terms of management strategies for chronic diseases by National Health Systems (NHS), particularly in terms of preventive care, with the result of underdiagnosis and undertreatment of these conditions and a consequent detrimental impact on public health and socio-economic burden, as underlined by the WHO [3]. To better understand how the choice of behaviours associated with a negative lifestyle is crucial in the relationship with chronic diseases responsible for the greatest number of deaths, it is useful to refer to the 3-four-50 model [15]. This model emphasizes that the 3 main components of the modern lifestyle, such as smoking, poor diet, and low levels of PA, contribute to the incidence, severity, and economic burden, of the four most relevant diseases for the current generation, such as cancer, cardiovascular and cerebrovascular diseases, type 2 diabetes, and pulmonary diseases, responsible for over 50% of deaths all around the world. This alarming scenario clearly requires promoting successful strategies to hinder the growing prevalence of negative lifestyle-related diseases. If we go beyond this obvious observation, considering that everyone knows that smoking habits, alcoholism, unhealthy diet, and a sedentary lifestyle are dangerous for personal wellbeing, it is necessary to look for the behavioural aspects that lead people to adopt lifestyles that threaten their lives. People usually think of the price to pay for wellbeing (increased PA, radical change in eating habits) as immediate, while the short-term benefit of this approach usually goes unnoticed [16]. Moreover, people are overly optimistic about their ability to take corrective action [17]. In this study we defined personalized path of PA by classifying different activities by their intensity, using the MET as a reference. MET is a largely adopted unit of measure of the PA. One MET is the rate of energy expenditure while sitting at rest. It is taken by convention to be an oxygen uptake of 3.5 ml per kilogram of body weight per minute or 1 kcal/kg/h. Moderate-intensity PA refers to the PA that is performed at 3.0–5.9 times the intensity of rest (3.0–5.9 METs). Vigorous-intensity PA refers to PA that is performed at 6.0 or more times the intensity of rest (> 6 METs) [10].

According to WHO guidelines adults should do at least 150 min of moderate-intensity aerobic PA or at least 75 min of vigorous-intensity aerobic PA throughout the week or an equivalent combination of moderate- and vigorous-intensity activity. For additional health benefits, adults should increase their moderate-intensity aerobic PA to 300 min per week or engage in 150 min of vigorous-intensity aerobic PA per week [10]. Moreover, it is recommended to start with a small amount of PA and then progressively increase volume of PA to avoid possible excessive tiredness or musculoskeletal pain [18].

These statements are consistent with the guidelines proposed by the U.S. Department of Health and Human Services that say that:” When adults do the equivalent of 150 min of moderate-intensity aerobic activity each week, the benefits are substantial. These benefits include lower risk of premature death, coronary heart disease, stroke, hypertension, type 2 diabetes, and depression. Not all health benefits of PA occur at 150 min a week. As a person moves from 150 min a week toward 300 min (5 h) a week, he or she gains additional health benefits. Additional benefits include lower risk of colon and breast cancer and prevention of unhealthy weight gain.” [19]. Moreover, they say that: “Aerobic PA should preferably be spread throughout the week. Research studies consistently show that activity performed on at least 3 days a week produces health benefits. Spreading PA across at least 3 days a week may help to reduce the risk of injury and avoid excessive fatigue.”

Many efforts have been devoted by WHO for promoting PA. The Global Action Plan on Physical Activity 2018–2030 has the scope to ensure safe and skillful access to facilities for PA improving individual and community health and contributing to the social, cultural, and economic development of all nations. The objective declared for 2030 is a relative reduction for adults and adolescent of 15% of global prevalence of physical inactivity [20]. In the past, several educational campaigns have been promoted for supporting behavior change and PA with mixed results [21–25]. However, most of these interventions promoted through several different media to perform physical activities, simply informing about their benefits. Our proposal acting on a playful aspect based on the achievement of specific physical goals, could improve the compliance of these subjects. Furthermore, recent COVID-19 pandemic has stressed this issue, increasing the amount of people performing insufficient PA during the quarantine. Physical inactivity and home working have increased the incidence of several painful musculoskeletal condition [26]. People limited their daily activity, following sedentary behaviors. Moreover, doing home exercise could be difficult for some individuals due to the incorrect use of IT available for encouraging PA at home [27]. The limitations due to COVID-19 have a significant impact on mental and emotional health, with dramatic effect on psychological well-being [28]. Promoting energy expenditure by recording the time spent and intensity used for common daily activities, as proposed by our algorithm, could encourage an appropriate lifestyle and be keystone to overcome these issues.

Our conceptual framework can be useful to facilitate local, regional, national, or international projects aimed at promoting PA on evidence-based basis. The idea is also to provide some “rewards” to people who reach the personalized targets of PA (e.g., discounts card to be used when buying sports stuff). The algorithm developed according to our conceptual framework will also be able to determine the different types and the duration of exercises needed to reach the personalized goals (so that individuals will be also able to choose what activity they want to practice each day according to the available time and personal feelings). The ultimate goal is to provide a tool that can support people to stay healthy by fostering their PA not in a “generic” manner but based on a scientific path (to achieve that, we proposed the computation of METs on the basis of individual answers to a validated questionnaire). As future perspective we will proceed to propose the algorithm as an operational tool for the implementation of preventive projects at community level and experimental data will be available so that an on-field validation of the tool will be possible. Therefore, in this paper we have described methodological details as first conceptual step of a larger project to enhance PA participation.

However, our proposal lacks assessment about participants’ motivation and cultural/psychological determinants of being engaged in PA. This issue could limit the compliance to the use of the application by consumers. Moreover, although the project is directed mainly to the general healthy population, it is likely that people over 50 years are affected by some disease. In this case, the disease, even if identified, must not reduce the independence in daily life, nor require any type of drug therapy.

Considering that NHS must deal with the progressive reduction of available resources, the realization of evidence-based public health programs aimed at promoting lifestyle interventions that are cost-effective and feasible in community settings is a crucial issue for researchers, health managers, and policymakers. Behaviorally based strategy addressing participation to PA programs have been demonstrated to be cost-effective compared to center-based exercise programs [29]. In this context, our proposal might be easily accessible, cost-effective, and reliable to allow both measuring and enhancing participation of large population to PA programs in community settings. This paper might be used also as a framework for implementing the development of other community-based lifestyle interventions. However, we provided only a theoretical model that requires to be implemented in further research.

## Conclusion

In this study we provided evidence-based standardised profiles and personalized goals of PA to be used through a dedicated platform that take into account people needs according to basal levels of PA thus increasing self-efficacy. Even if our proposal requires to be further implemented, this web-based resource would be a reliable method for increasing people adherence to adequate levels of PA.

## Data Availability

Statistical code and dataset freely available upon reasonable request to Prof. Giovanni Iolascon, by using the following email address: giovanni.iolascon@unicampania.it.

## Abbreviations

NCDs: Non-Communicable Diseases
PA: Physical activity
METs: Metabolic equivalents
GPPAQ: General Practice Physical Activity Questionnaire
PG: Personal Goal
St: Standard Goal
GAw: Global amount of MET set over the week
CVw: Level of coverage for the week
Dw: Global distribution of the MET over the week
Fw: Fitness of the distribution
CONw: Level of standard concentration
PERFw: The amount of METs performed in the week
UNCw: The relative amount of METs not accomplished in the current week respect on the scheduled METs
NHS: National Health Systems
